# IL-4 as a Repurposed Biological Drug for Myocardial Infarction through Augmentation of Reparative Cardiac Macrophages: Proof-of-Concept Data in Mice

**DOI:** 10.1038/s41598-017-07328-z

**Published:** 2017-07-31

**Authors:** Yusuke Shintani, Tomoya Ito, Laura Fields, Manabu Shiraishi, Yuki Ichihara, Nobuhiko Sato, Mihai Podaru, Satoshi Kainuma, Hiroyuki Tanaka, Ken Suzuki

**Affiliations:** 1 William Harvey Research Institute, Barts and The London School of Medicine, Queen Mary University of London, London, United Kingdom; 2 Cardiovascular Surgery, Kurume University, Fukuoka, Japan

**Keywords:** Cardiovascular diseases, Heart failure

## Abstract

Recent research has shown that reparative (alternatively activated or M2) macrophages play a role in repair of damaged tissues, including the infarcted hearts. Administration of IL-4 is known to augment M2 macrophages. This translational study thus aimed to investigate whether IL-4 administration is useful for the treatment of myocardial infarction. Long-acting IL-4 complex (IL-4c; recombinant IL-4 mixed with anti-IL-4 monoclonal antibody as a stabilizer) was administered after coronary artery ligation in mice. It was observed that IL-4c administration increased accumulation of CD206^+^F4/80^+^ M2-like macrophages predominantly in the injured myocardium, compared to the control. Sorted cardiac M2-like macrophages highly expressed wide-ranging tissue repair-related genes. Indeed, IL-4c administration enhanced cardiac function in association with reduced infarct size and enhanced tissue repair (strengthened connective tissue formation, improved microvascular formation and attenuated cardiomyocyte hypertrophy). Experiments using *Trib1* ^*−/−*^ mice that had a depleted ability to develop M2 macrophages and other *in-vitro* studies supported that these IL-4-mediated effects were induced *via* M2-like macrophages. On the other hand, when administered at Day 28 post-MI, the effects of IL-4c were diminished, suggesting a time-frame for IL-4 treatment to be effective. These data represent proof-of-concept of efficacy of IL-4 treatment for acute myocardial infarction, encouraging its further development.

## Introduction

Myocardial infarction (MI) remains a major cause of death and disability worldwide. While early survival after acute MI has been improved by recent progress in interventional and pharmacological therapy, this resulted in an increasing number of patients who develop post-MI chronic heart failure (ischemic cardiomyopathy)^1^. A crucial process for survival of MI is immediate and long-lasting reinforcement of the fragile infarcted myocardium with the formation of solid connective tissues^2^. This local fibrotic scar formation is essential to prevent cardiac rupture. In addition, even when the infarcted heart escapes the rupture, the surviving myocardium undergoes cellular and molecular adverse remodeling, which is underpinned by chronic local inflammation, persistent ischemia and excessive mechanical overload^3^. This progressively deteriorates the viable myocardium through inducing apoptosis and pathological hypertrophy of surviving cardiomyocytes as well as pathological interstitial fibrosis in the border and remote areas of the infarct, leading to progression of cardiac dilatation and dysfunction. Therefore, improvement of formation of solid fibrotic tissues in the infarct as well as prevention and/or treatment of adverse ventricular remodeling are the key to improvement of the prognosis of MI patients. However, due to insufficient efficacy of currently available therapies, many of the patients ultimately go on to develop end-stage heart failure.

Distinct types of monocytes and macrophages play a role in initiation, maintenance, and resolution of myocardial inflammation post-MI^4–8^, which control cardiac repair and adverse ventricular remodeling. With a peak around Day 3 post-MI, inflammatory signals from the heart recruit monocytes from the circulation (*i.e*. from bone marrow and spleen), which predominantly differentiate and mature into classically-activated, pro-inflammatory (M1) Ly-6C^hi^CD206^−^ macrophages^4^. These macrophages degrade and remove necrotic debris and apoptotic cells, which is a necessary preparation for tissue repair. The classification of macrophages into M1 and M2 phenotypes may be an oversimplification but is still helpful when primarily discussing the function of the macrophage subtypes. Subsequently, along with the reduction of the increased M1 macrophages, the number of reparative, anti-inflammatory (alternatively activated or M2) Ly-6C^lo^CD206^+^ macrophages increases in the heart, with a peak at Day 5–7 post-MI^4–8^. These cells play a role in the resolution of acute inflammation as well as facilitation of prompt extracellular matrix synthesis by activating fibroblasts, together resulting in the formation of fibrotic scar tissues to reinforce the vulnerable ventricular wall that had lost cardiomyocytes^5–9^. M2 macrophages also appear to be involved in other self-repair systems, including induction of neovascular formation in ischemic tissues and attenuation of post-MI adverse ventricular remodeling^5–9^. On the other hand, recent research has shown that cardiac macrophages have another origin in addition to the bone marrow and spleen. Cardiac macrophages present in the normal heart, which are called “cardiac resident macrophages”, are stemmed from the yolk-sac prenatally and persist through the adulthood with self-renewal *in-situ* without input from circulating monocytes^6, 10^. The majority of cardiac resident macrophages have an M2 macrophage phenotype, expressing a group of tissue repair-related genes, and are considered to contribute to the maintenance of myocardial homeostasis under the ordinary environment^11^.

Augmentation of cardiac M2 macrophages will, therefore, be a promising therapeutic approach for the treatment of MI. This treatment will encourage formation of more solid scar tissue, resolution of acute inflammation, and attenuation of post-MI adverse ventricular remodeling. Previous studies have reported several methods to increase cardiac M2 macrophages, including intramyocardial injection of microparticles loaded with fibroblast growth factor-2 with hepatocyte growth factor^12^, intravenous injection of phosphatidylserine-presenting liposomes^13^, intramyocardial injection of FGF-9^14^, administration of superagonistic CD28-specific monoclonal antibodies^15^, and intramyocardial transplantation of mesenchymal stem cells^16^. However, these are technically challenging and none of them has been established in the clinical arena yet. Development of a simpler and effective method to augment cardiac M2 macrophages is of great value to translate the M2-macrophage-based therapy to the bedside.

Interleukin 4 (IL-4) is a Th2 cytokine that regulates multiple biological functions^17, 18^. Particularly, it is reported that IL-4 drives differentiation of monocytes/macrophages to M2 phenotypes and increases proliferation of M2 macrophages *in situ* ^17, 19^. We recently reported that external administration of IL-4 improved myocardial repair and enhanced function of damaged hearts^9^; however, this was a finding in a model which is not clinically-relevant (injection of IL-4 before the onset of MI). It remains unknown whether administration of IL-4 post-MI, in which a range of cytokines and growth factors vigorously influence in a mutually-interacting manner, is able to augment cardiac M2-like macrophages and/or improve post-MI cardiac function. Also, it is likely that the efficacy of the IL-4 treatment is affected by the timing of the treatment (i.e., treatment at acute phase versus late phase post-MI). This translational research therefore aims to establish pre-clinical data showing the efficacy of IL-4 administration in clinically relevant models of the treatment of MI. Repurposing IL-4, which has already been shown to be safe in clinical trials of cancer treatment^20–24^, will represent a “low-risk high-return” development of a new biological drug for the treatment of MI.

## Results

### Increased cardiac M2-like macrophages post-MI by IL-4c treatment

We first investigated whether and how IL-4 administration after the onset of MI would increase M2 macrophages in the heart. IL-4 complex (IL-4c; a mixture of 5 μg recombinant mouse IL-4 and 25 µg anti-IL-4 monoclonal antibody dissolved in PBS; IL-4 group) or PBS only (PBS group) was injected intraperitoneally at 1 hour after left coronary artery ligation in adult C57BL/6 mice. Mixture of recombinant IL-4 with an anti-IL-4 antibody is known to stabilize the IL-4 protein and expand its *in-vivo* half-life from 30 minutes (sole protein) to over 24 hours^17, 25^.

**Figure 1:**
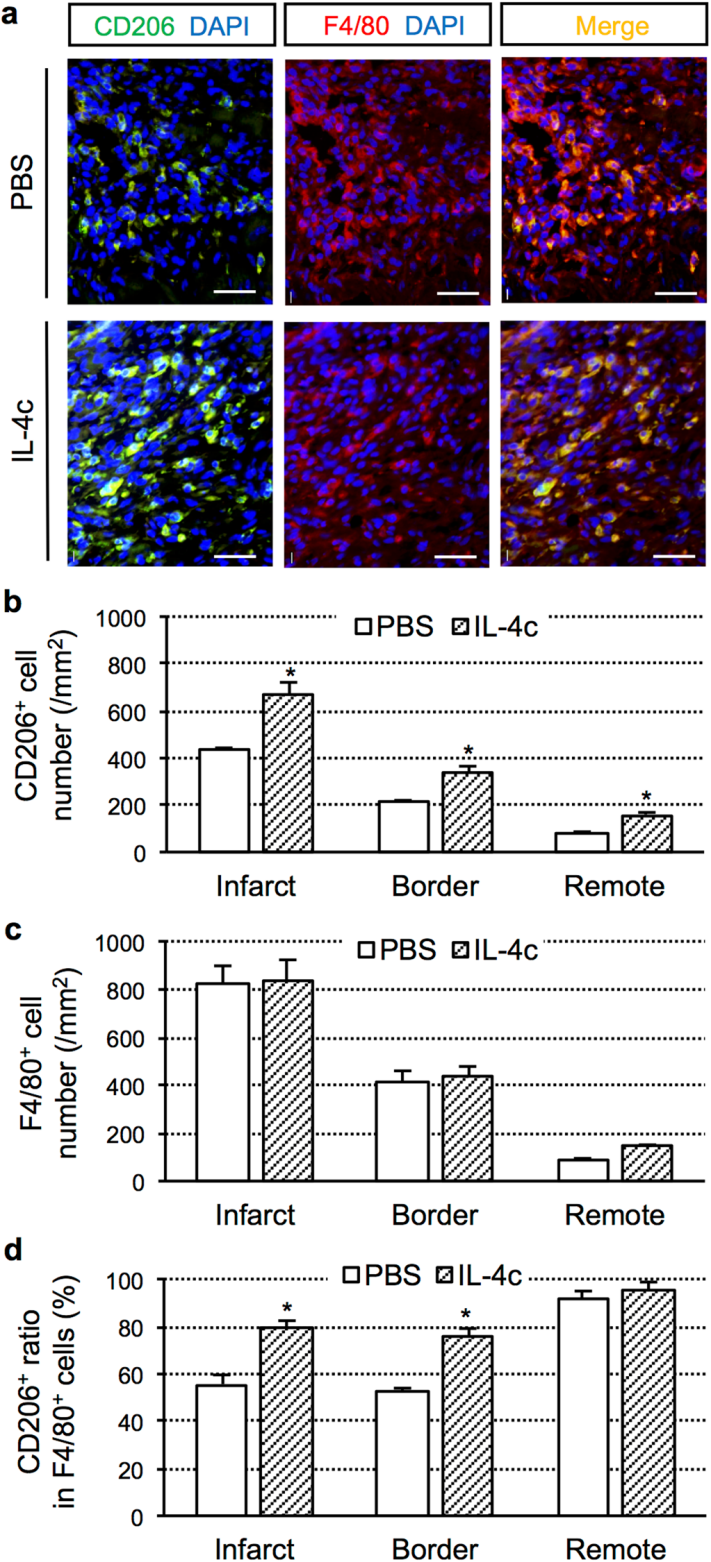
Increased cardiac M2-like macrophages post-MI by IL-4c treatment. Following coronary artery ligation, IL-4c (IL-4 group) or PBS alone (PBS group) was injected intraperitoneally to the mice. The hearts at Day 7 post-treatment were stained for CD206 and F4/80 with nuclear staining using DAPI. (**a**) Representative pictures from the infarct area are presented (see Supplementary Fig. S1 for the pictures of other areas). Scale bars, 100 μm. (**b**) The numbers of CD206^+^ cells in each area of the MI heart were counted. (**c**) The numbers of F4/80^+^ cells in each area were counted. (**d**) The ratios of CD206^+^ cells in F4/80^+^ cells in each area were calculated. *N* = 5 hearts in each group; *P < 0.05 versus the PBS group.

Immunohistolabeling demonstrated that IL-4c treatment achieved a more extensive increase of CD206^+^ cells at Day 7 post-MI, compared to the PBS control, with the most evident increase being seen in the infarct area (Fig. 1a,b and Supplementary Fig. S1). The number of CD206^+^ cells in the normal mouse heart was 88.1 ± 4.5 cells/mm^2^ (*N* = 6 hearts). CD206^+^ cells displayed a typical macrophage morphology, and more than 95% of CD206^+^ cells were positive for F4/80 in all myocardial areas in both groups (Fig. 1a and Supplementary Fig. S1), suggesting their identity as M2-like macrophages. In contrast, the total numbers of F4/80^+^ macrophages in each area were equivalent between the groups (Fig. 1a,c). Consequently, the ratio of CD206 positivity in F4/80^+^ macrophages in the injured myocardium were higher in the IL-4c group compared to the PBS group (80% versus 50%; Fig. 1d), indicating that IL-4c treatment drove the polarization of cardiac macrophages toward the M2 phenotype. At Day 28 after MI with treatment, the numbers of CD206^+^ cells in the infarct and border myocardium remained greater in the IL-4c-treated hearts compared to those of the PBS control (Supplementary Fig. S2).

### Highly expressed tissue repair-related genes in cardiac CD206^+^F4/80^+^ cells

**Figure 2:**
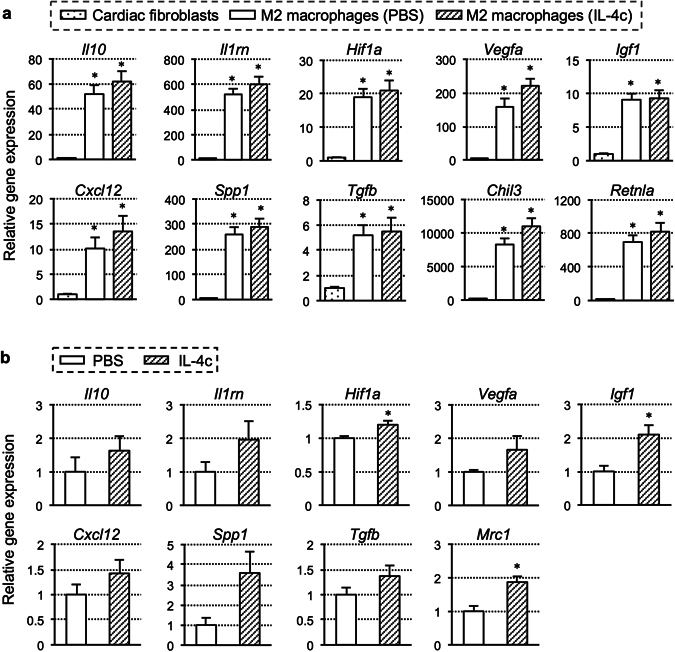
Expression profiles of cardiac CD206^+^F4/80^+^ cells and post-MI myocardium. (**a**) CD206^+^F4/80^+^ cells were sorted from the heart in the IL-4c and PBS groups and subjected to real-time PCR. Cardiac fibroblasts were also isolated and measured for comparison. Expression of each gene in fibroblasts was assigned to 1.0. Cells from 4 different hearts in each group (*N* = 4) were studied; *P < 0.05 versus cardiac fibroblasts. (**b**) Expression of genes in the LV free wall myocardium (including border and infarct areas) from each group was assessed by real-time PCR. Expression in the PBS group was assigned to 1.0. *N* = 5 in each group; *P < 0.05 versus the PBS group.

We isolated CD206^+^F4/80^+^ cells using fluorescence-activated cell sorting from the mouse heart at Day 7 post-MI with the administration of IL-4c or PBS. Quantitative PCR screening revealed that CD206^+^F4/80^+^ cells collected from either group expressed high levels of genes that are related to anti-inflammation, angiogenesis, tissue repair and regeneration connective tissue formation, including *Il10, Il1rn, Hif1a, Vegfa, Igf1, Cxcl12, Spp1* and *Tgfb*, (Fig. 2a). These cells also expressed high levels of M2 macrophage marker genes including *Chil3 (Ym1)* and *Retnla* (*Fizz1)*. Some of these genes showed a tendency of more extensive up-regulation in the IL-4c group, though not statistically significant. Corresponding to this observation *in vitro*, expression of *Igf1* and *Hif1a*, in addition to *Mrc1* (*Cd206*), in the post-MI myocardial tissue (including the infarct and border areas) was significantly upregulated in the IL-4c group, compared to the PBS group (Fig. 2b). Other reparative genes also appeared to show an upregulating tendency in the IL-4c group, but this was not statistically significant.

### Enhanced repair of the damaged myocardium by IL-4c treatment

**Figure 3:**
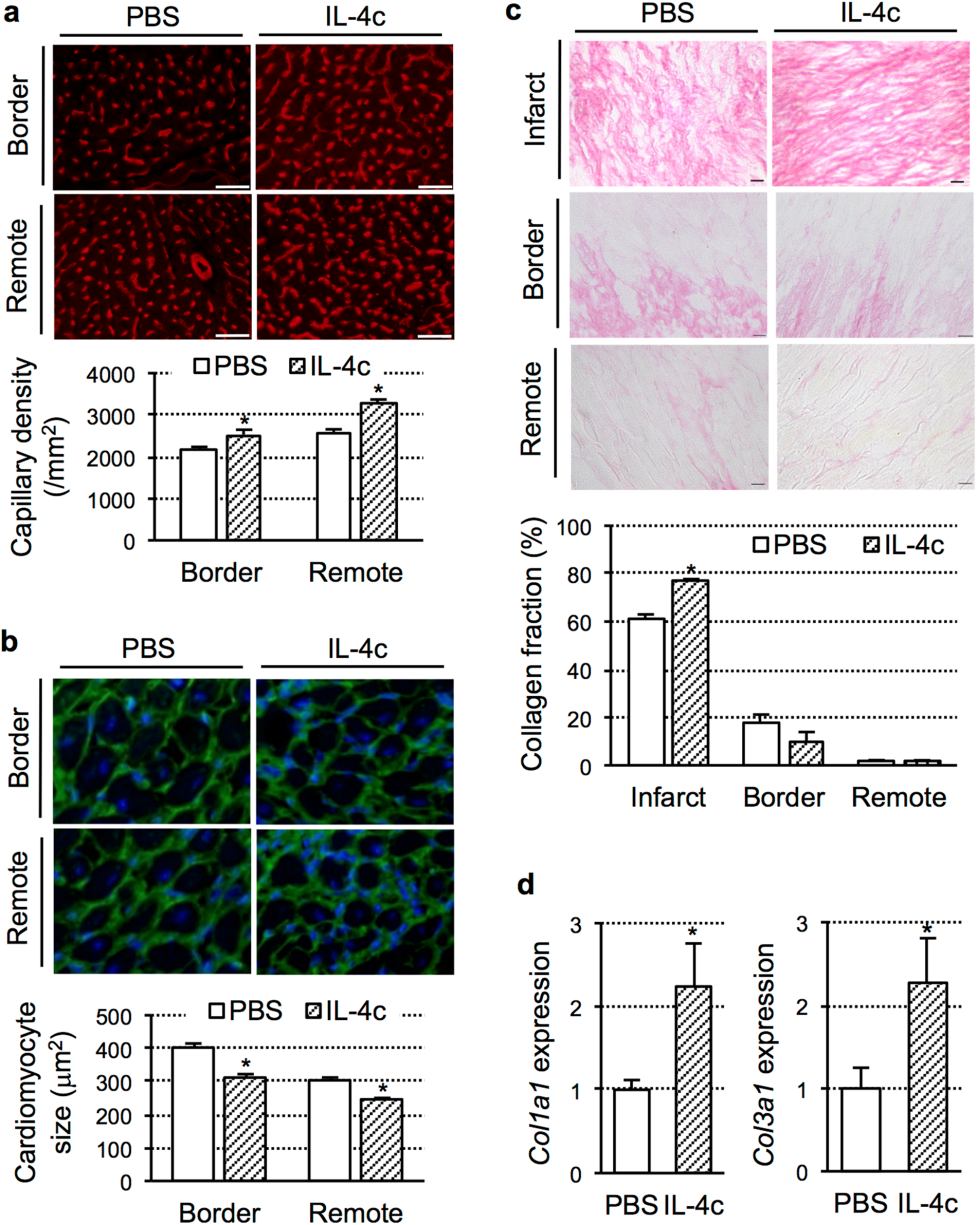
Enhanced myocardial repair by IL-4c treatment. (**a**–**c**) The hearts at Day 28 after treatment in each group were stained with Isolectin B4 (**a**), Wheat Germ Agglutinin with DAPI nuclear staining (**b**) and Picrosirius red (**c**). Scale bars, 50 μm. With these samples, the capillary density, short-axis area of cardiomyocytes, collagen volume fractions in each myocardial area were measured/calculated and shown in the graphs. *N* = 5 hearts in each group; *P < 0.05 versus the PBS group. (**d**) Expression of collagen genes in the LV free wall myocardium was assessed by real-time PCR. *N* = 5 hearts in each group; *P < 0.05 versus the PBS group.

Histological studies demonstrated that above-observed up-regulation of anti-inflammatory and tissue repair-related genes in the heart by IL-4c treatment was indeed correlated to enhanced tissue repair and reverse remodeling of the failing myocardium post-MI. Capillary density in the viable (remote and border) myocardial areas assessed by isolectin B4 staining was greater in the IL-4c group compared to the PBS group (Fig. 3a). Staining for wheat germ agglutinin elucidated that hypertrophy of surviving cardiomyocytes observed in the border and remote areas of the PBS group was attenuated in the IL-4c group (Fig. 3b). Cardiomyocyte size in the normal mouse heart was 233.3 ± 14.9 μm^2^ (*N* = 40 cells from 4 hearts). In addition, picrosirius red staining showed that the IL-4c group developed denser connective tissues in the infarct (local fibrosis), with a higher collagen volume fraction, compared to the PBS control (Fig. 3c). In correspondence to this, the IL-4c treated heart presented increased expression of collagen gene, compared to the PBS control (Fig. 3d). By contrast, pathological interstitial fibrosis in the remote and border areas, which is one of the major events of post-MI adverse ventricular remodeling, was not exaggerated by the IL-4c treatment (Fig. 3c).

### Improved post-MI cardiac function and structure by IL-4c treatment

**Figure 4:**
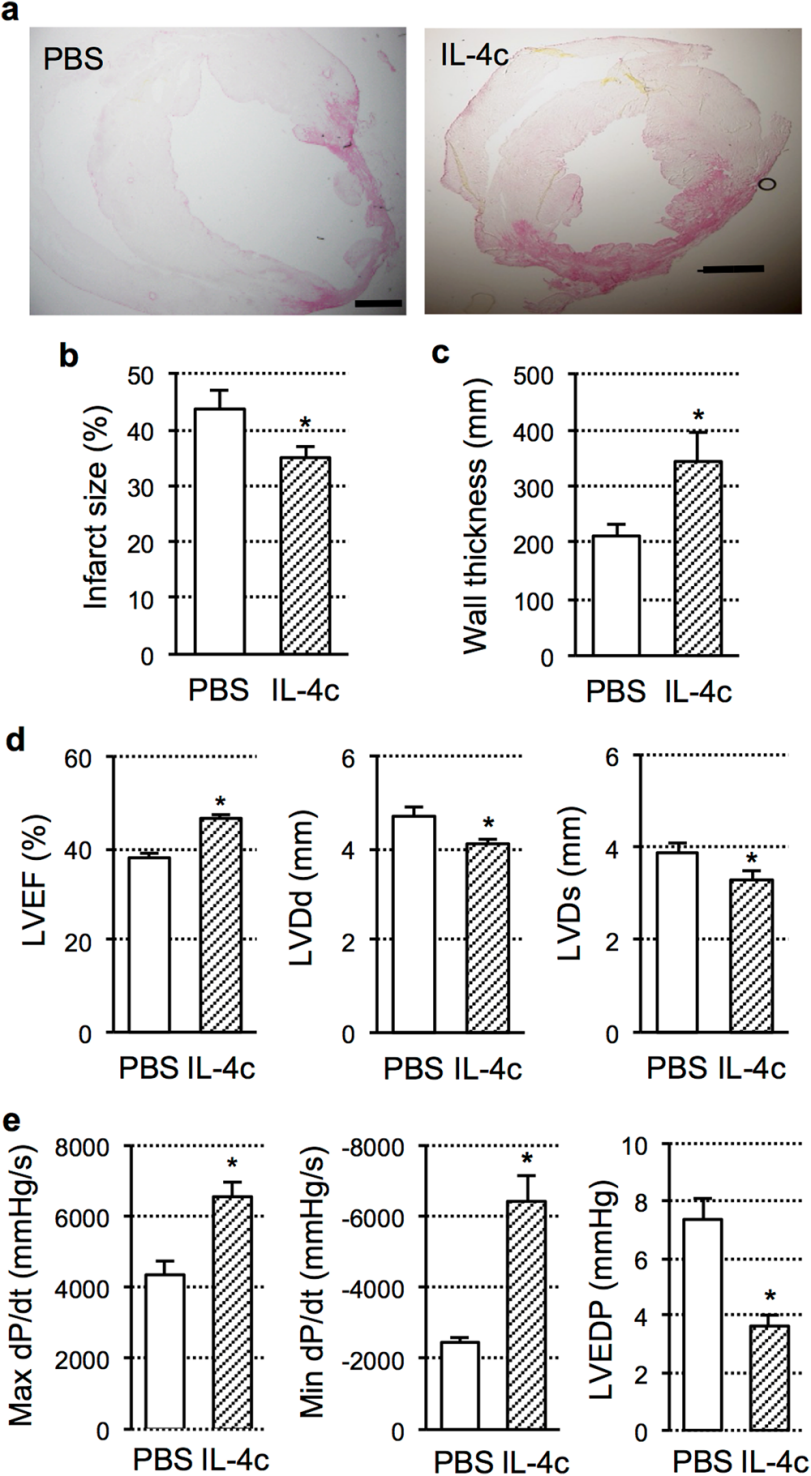
Improved cardiac structure and function by IL-4c treatment. (**a**–**c**) The hearts at Day 28 after treatment were stained with Picrosirius red (**a**), and infarct size (**b**) and thickness of the infarcted myocardium (**c**) was measured. Scale bars, 300 μm. *N* = 5 in each group; *P < 0.05 versus the PBS group. (**d**) Echocardiographic measurements were performed at Day 28 after treatment. LVEF; left ventricular ejection fraction, LVDd/LVDs; left ventricular diastolic/systolic dimension. *N* = 10 in each group; *P < 0.05 versus the PBS group. (**e**) Catheterization was conducted at Day 28 after treatment. LVEDP; left ventricular end-diastolic pressure. *N* = 5 in each group, *P < 0.05 versus the PBS group.

Underpinned by myocardial repair shown above, IL-4c treatment certainly improved structure and function of the heart post-MI. Histological examinations elucidated that the infarct size was reduced by IL-4c treatment compared to PBS injection (Fig. 4a,b). Also, the thickness of the infarcted left ventricular (LV) walls was increased (Fig. 4a,c). Echocardiography at Day 28 after treatment demonstrated that LV ejection fraction was improved in the IL-4c group, along with the LV dimensions (both end-diastole and end-systole) being reduced (Fig. 4d and Supplementary Table S1). Corresponding to the echocardiographic data, cardiac catheterization showed that systemic and diastolic LV function, in terms of maximum and minimum LV dP/dt, was improved in the IL-4c group compared to the PBS group (Fig. 4e and Supplementary Table S2). In addition, increased LV end-diastolic pressure in the PBS group was normalized in the IL-4c group. Thus, it was confirmed that IL-4c treatment enabled to offer therapeutic benefits, indicating a great potential of this biological drug as an effective treatment for acute MI.

### Mechanisms for the improved connective tissue formation in the infarcts by IL-4

**Figure 5:**
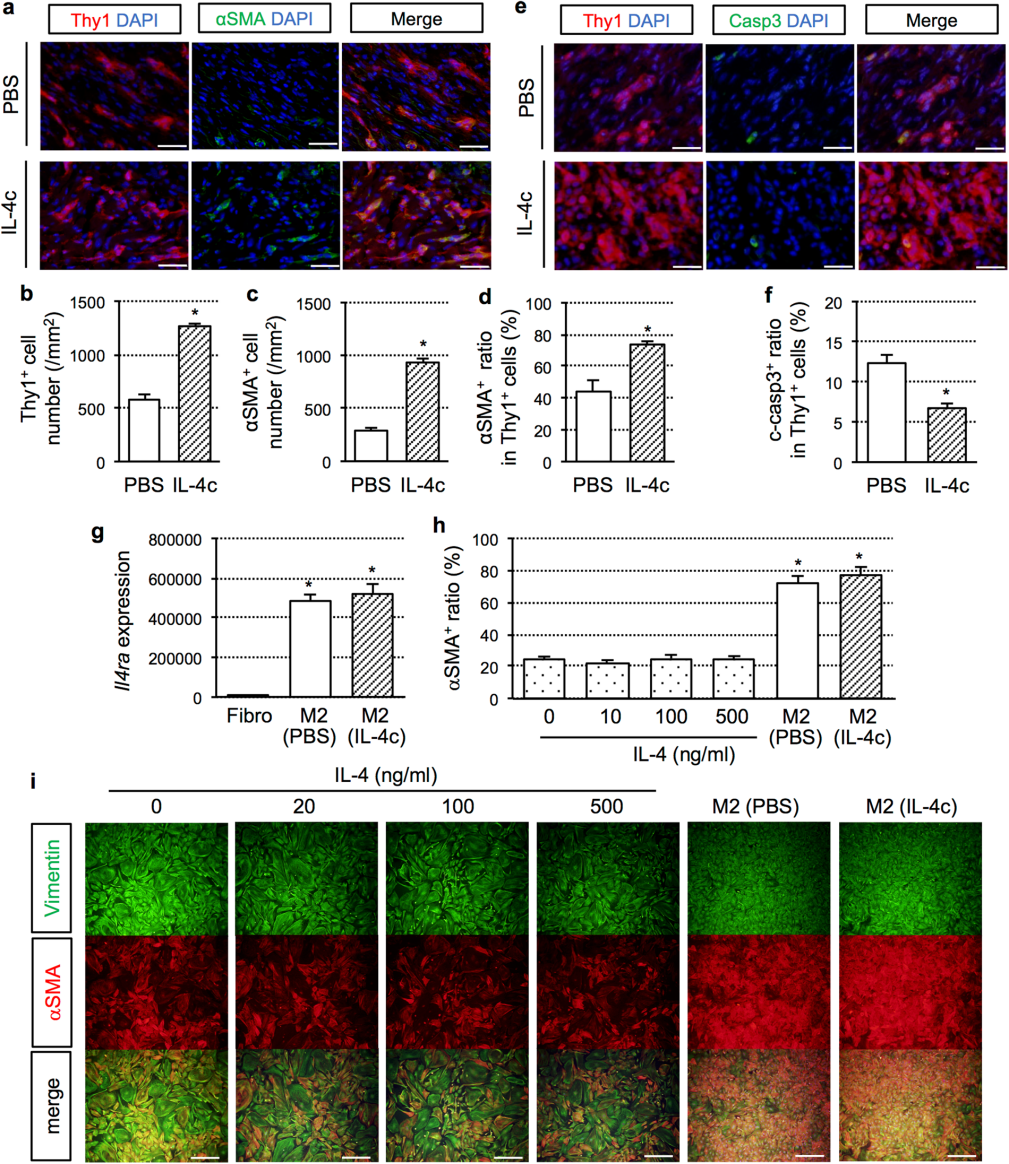
Enhanced activation of cardiac fibroblasts by IL-4c treatment. (**a**) The hearts at Day 7 after treatment were stained for Thy1 and αSMA with DAPI nuclear staining. Scale bars, 50 μm. (**b**–**d**) The number of Thy1^+^ (**b**) and αSMA^+^ cells (**c**), and the ratio of αSMA^+^ cells in Thy1^+^ cells (**d**) in the infarct area are shown in the graphs. Please see Fig. S3 for the data in the border and remote areas. *N* = 5 different hearts in each group. *P < 0.05 versus PBS group. (**e**) The hearts at Day 7 after treatment were stained for Thy1 and cleaved caspase-3 with DAPI nuclear staining. Scale bars, 50 μm. (**f**) The ratios of cleaved caspase-3^+^ cells in Thy1^+^ cells in the infarct area are shown in the graph. *N* = 5 in each group. *P < 0.05 versus PBS group. (**g**) CD206^+^F4/80^+^ cells sorted from the heart in the IL-4c and PBS groups [M2 (IL-4c) and M2 (PBS), respectively], in addition to cardiac fibroblasts (Fibro), were subjected to real-time PCR for *Il4ra*. Expression in fibroblasts was assigned to 1.0. *N* = 5 in each group; *P < 0.05 versus cardiac fibroblasts. (**h** and **i**) Primary cardiac fibroblasts were cultured *in vitro* with recombinant IL-4 or with CD206^+^F4/80^+^ cells sorted from the heart in the IL-4c and PBS groups [M2 (IL-4c) and M2 (PBS), respectively], and then stained for vimentin and αSMA. The ratios of αSMA^+^ cells in vimentin^+^ cells are shown in the graph. *N* = 6 in each group. *P < 0.05 versus all recombinant IL-4 *in-vitro* administration groups. Representative pictures are shown in (**i**).

Formation of the firm infarct scar, which helps maintain the configuration of the fragile infarcted myocardium post-MI, is a critical determinant of the outcome of MI^1, 2^. We thus explored the cellular changes responsible for the improved fibrotic tissue formation by IL-4c treatment. Immunohistofluorescence demonstrated that the numbers of Thy1^+^ fibroblasts and αSMA^+^Thy1^+^ myofibroblasts in the infarct area at Day 7 were both larger in the IL4c group compared to the PBS group (Fig. 5a–c). The ratio of αSMA positivity in Thy1^+^ cells was also higher in the IL-4c group (Fig. 5d). In addition, apoptosis of cardiac fibroblasts was attenuated in the IL-4c group (Fig. 5e,f). These data suggested that IL-4c treatment amplified activation as well as protection of cardiac fibroblasts in the infarcted myocardium, leading to strengthened connective tissue formation. In contrast, the number of cardiac fibroblasts was not increased in the remote and border areas by IL-4c treatment (Supplementary Fig. S3), corresponding to the above result (Fig. 3c) showing unchanged interstitial fibrosis in these areas by the IL-4c treatment.

We then investigated the mechanism responsible for the enhanced cardiac fibroblast activation by IL-4c treatment. Several pieces of evidence below implied that the IL-4-mediated activation of fibroblasts would be developed through IL-4-increased M2-like macrophages, and a direct effect of IL-4 on fibroblasts was unlikely. Firstly, gene expression of the main receptor of IL-4, *Il4ra*, in isolated primary cardiac fibroblasts was 1/10^5^-fold lower compared to that in cardiac CD206^+^F4/80^+^ cells sorted from infarcted hearts (Fig. 5g). Secondly, an addition of up to 500 ng/ml IL-4 did not increase activation (transformation to αSMA^+^ myofibroblasts) of primary cardiac fibroblasts *in vitro* (Fig. 5h,i). While in contrast, co-culture with CD206^+^F4/80^+^ M2-like macrophages isolated from the infarcted heart markedly activated fibroblasts to αSMA^+^ myofibroblasts. Thirdly, cardiac CD206^+^F4/80^+^ M2-like macrophages isolated from the post-MI heart expressed higher levels of major pro-fibrotic factors, including *Tgfb* and *Spp1* ^2, 26^ (Fig. 2a), compared to primary cardiac fibroblasts, indicating an ability of cardiac M2 macrophages to activate cardiac fibroblasts post-MI.

### Cancelled effects of IL-4c treatment in *Trib1*^**−/−**^ mice that showed depleted function to develop cardiac M2-macrophages post-MI

**Figure 6:**
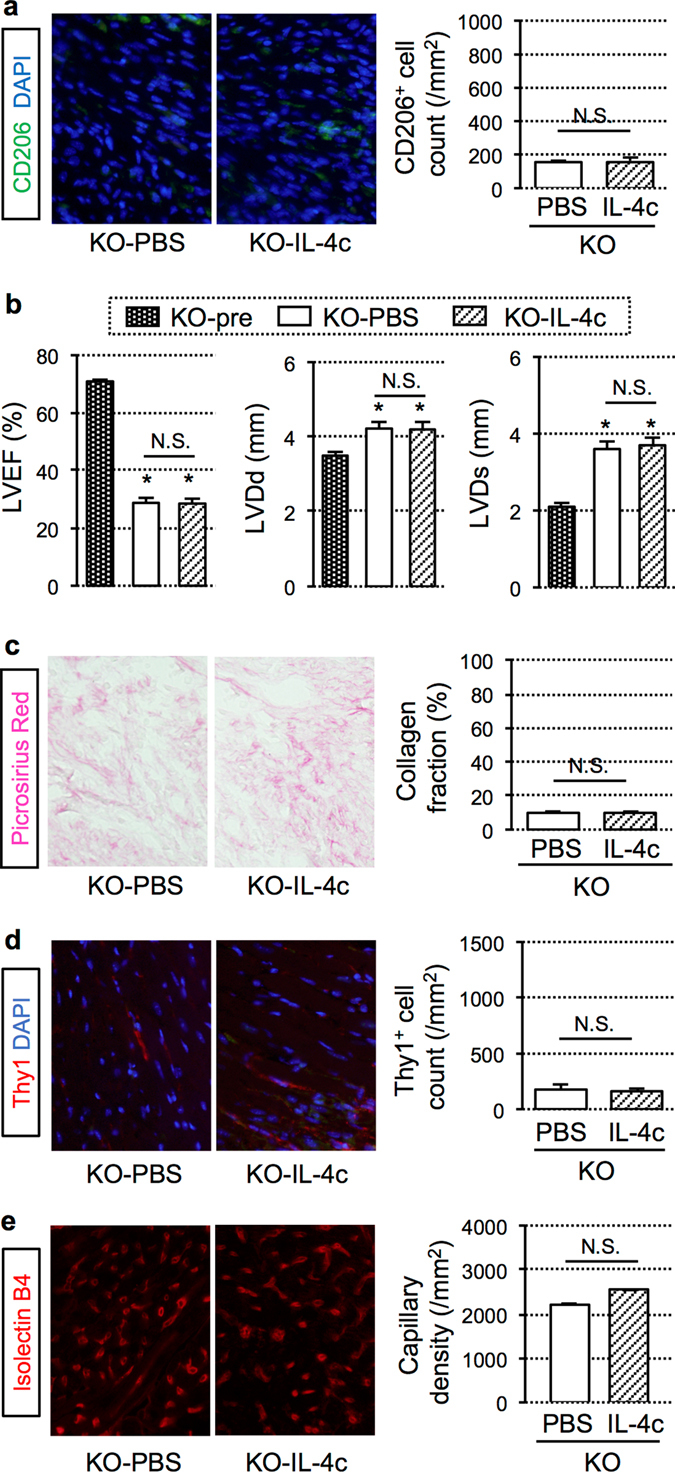
Cancelled effects of IL-4c treatment in *Trib1* ^*−/−*^ mice that had depleted accumulation of cardiac M2-macrophages post-MI. IL-4c (KO-IL-4c group) or PBS alone (KO-PBS group) was injected intraperitoneally to the *Trib1* ^*−/−*^ mice at 20 minutes after coronary artery ligation. (**a**) The hearts at Day 7 post-treatment were stained for CD206 and DAPI. Representative pictures from the infarct area are presented. Scale bars, 100 μm. The numbers of CD206^+^ cells were counted and presented in the graph group. (**b**) Echocardiography were performed at Day 7 after treatment. *N* = 6 in each group; *P < 0.05 versus the KO-pre group (no-MI *Trib1* ^*−/−*^ mice). (**c**–**e**) The collected hearts in each group were stained with Picrosirius red (**c**), Thy1 (**d**), and Isolectin B4 (**e**). Scale bars, 50 μm. With these samples, collagen volume fraction in the infarct area, the number of Thy1^+^ fibroblasts in the infarct area, and the capillary density in the peri-infarct area were measured/calculated and shown in the graphs. *N* = 5 hearts in each group; *P < 0.05 versus the KO-PBS group.

To further ensure that above-shown IL-4c-mediated therapeutic benefits were induced *via* M2-like macrophages, rather than the direct effect of IL-4 on other types of cardiac cells (e.g. cardiac fibroblasts), additional studies were carried out using *Trib1* ^*−/−*^ mice^27^. *Trib1* ^*−/−*^ mice are known to possess an impaired ability to form M2-like macrophages but not other inflammatory cells, in the spleen, liver, lung, and adipose tissue. We observed that post-MI accumulation of cardiac M2-like macrophages in the infarcted myocardium was obviously eliminated in *Trib1* ^*−/−*^ mice (refer to wild-type mouse data in Fig. 1a–b). Notably, IL-4c treatment could not increase cardiac M2-macrophages in *Trib1* ^*−/−*^ mice (Fig. 6a). Echocardiography revealed that the post-MI cardiac dysfunction and dilatation in *Trib1* ^*−/−*^ mice were not improved by the IL-4c treatment (Fig. 6b). Histological studies demonstrated that the abilities of IL-4c treatment to strengthen the connective tissue formation through activation of cardiac fibroblasts, and enhance neovascular formation in the peri-infarct viable areas, which were detected in wild-type mice (Fig. 3), were eliminated in *Trib1* ^*−/−*^ mice that had abolished accumulation of M2-like macrophages post-MI (Fig. 6c–e).

### Behaviors of cardiac “resident” M2-like macrophages

**Figure 7:**
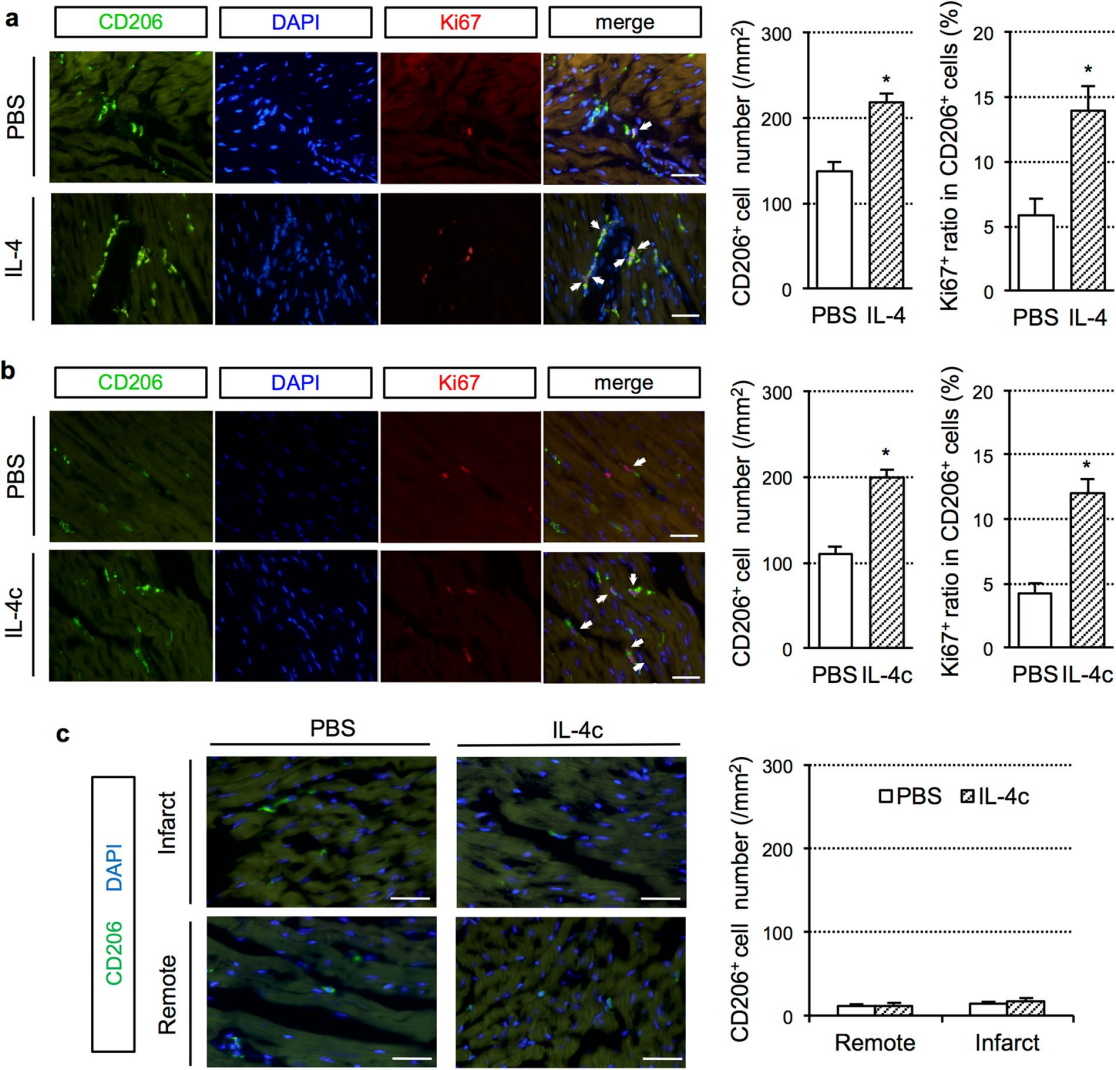
Source of increased cardiac M2-like macrophages by IL-4c treatment. (**a**) Viable heart slices were cultured with IL-4 (IL-4 group) or without IL-4 (PBS group) and subjected to immunolabeling for CD206 and Ki67 with DAPI nuclear staining. White arrows indicate CD206^+^Ki67^+^DAPI^+^ cells. The numbers of CD206^+^ and Ki67^+^ ratios in CD206^+^ cells were shown in the graphs. *N* = 5 different hearts in each group. *P < 0.05 versus PBS group. (**b**) Two days after intraperitoneally injection of IL-4c or PBS (control) into normal mice, the hearts were subjected to labeling for CD206, Ki67 and DAPI. White arrows indicate CD206^+^Ki67^+^DAPI^+^ cells. The numbers of CD206^+^ and Ki67^+^ ratios in CD206^+^ cells were counted and shown in the graphs. *N* = 5 hearts in each group. *P < 0.05 versus PBS group. (**c**) IL-4c or PBS was injected at 20 minutes after coronary artery ligation. At Day 1, the heart was subjected to immunofluorescence for CD206 with DAPI nuclear staining. The numbers of CD206^+^ cells were shown in the graphs. *N* = 5 hearts in each group.

We next explored the source(s) of increased cardiac M2-like macrophages by IL-4c treatment. It has been shown that there are at least two major origins for cardiac M2-like macrophages. Cardiac “resident” M2 macrophages existing in the normal heart are originated from the yolk sac at an embryonic stage, while bone marrow-derived monocytes recruit to the adult heart and differentiate into M2 macrophages in response to injury including MI^5–8, 10^. To test the effect of administration of recombinant IL-4 on cardiac “resident” M2 macrophages, 300 μm-thick viable heart slices were cut from the intact mouse heart and cultured in a dish *ex vivo* with or without 20 ng/ml IL-4. This model can exclude recruitment of CD206^+^ cells or their progenitors from the circulation. At 24 hours of culture, the IL-4-treated heart slices contained a larger number of CD206^+^ cells, which were also positive for F4/80, as compared to the control-cultured slices (Fig. 7a). In addition, CD206^+^ cells in the IL-4-treated heart slices displayed an elevated rate of Ki67 positivity than those in the control slices. These results were replicated in an *in-vivo* model; administration of IL-4c increased cardiac CD206^+^ cells, with an elevated Ki67^+^ ratio in these cells, in the normal mouse heart (Fig. 7b). These data suggest that external IL-4 can increase the number of M2-like macrophages in the heart *via* the local proliferation of cardiac “resident” M2-like macrophages. However, on the other hand, our immunohistological assessments demonstrated that cardiac CD206^+^ cells seldom existed throughout the heart at Day 1 post-MI (Fig. 7c), agreeing with the previous report^6^. Considering the healthy adult murine heart having a sizable (~100 cells/mm^2^) number of CD206^+^ cardiac “resident” M2-like macrophages as we have reported^9^, this result indicated that the majority (>90%) of these cells disappeared in 24 hours post-MI. Although the mechanism of this disappearance is uncertain, cardiac “resident” M2-like macrophages could be dead, change the phenotype (loss of CD206 expression) or/and exit from the heart in response to the MI insult. More-focused studies (*e.g*. using specific labeling of yolk-sac-derived resident cardiac macrophages) are needed to fully uncover the behavior of cardiac resident M2-like macrophages.

### Time-window for IL-4c administration to be effective to treat MI

Above findings consistently demonstrated that IL-4c administration was effective in the treatment of MI when conducted at an acute phase after the onset of MI. Our next question was whether this therapy would be effective when performed at a late phase post-MI (*i.e*. for the treatment of post-MI chronic heart failure). Due to the different myocardial conditions (*e.g*. acute or chronic inflammation, different frequency of circulating monocytes, immature or established fibrosis, *etc*.), the response of the heart to external IL-4 may be diverse between the acute and late phase post-MI.

**Figure 8:**
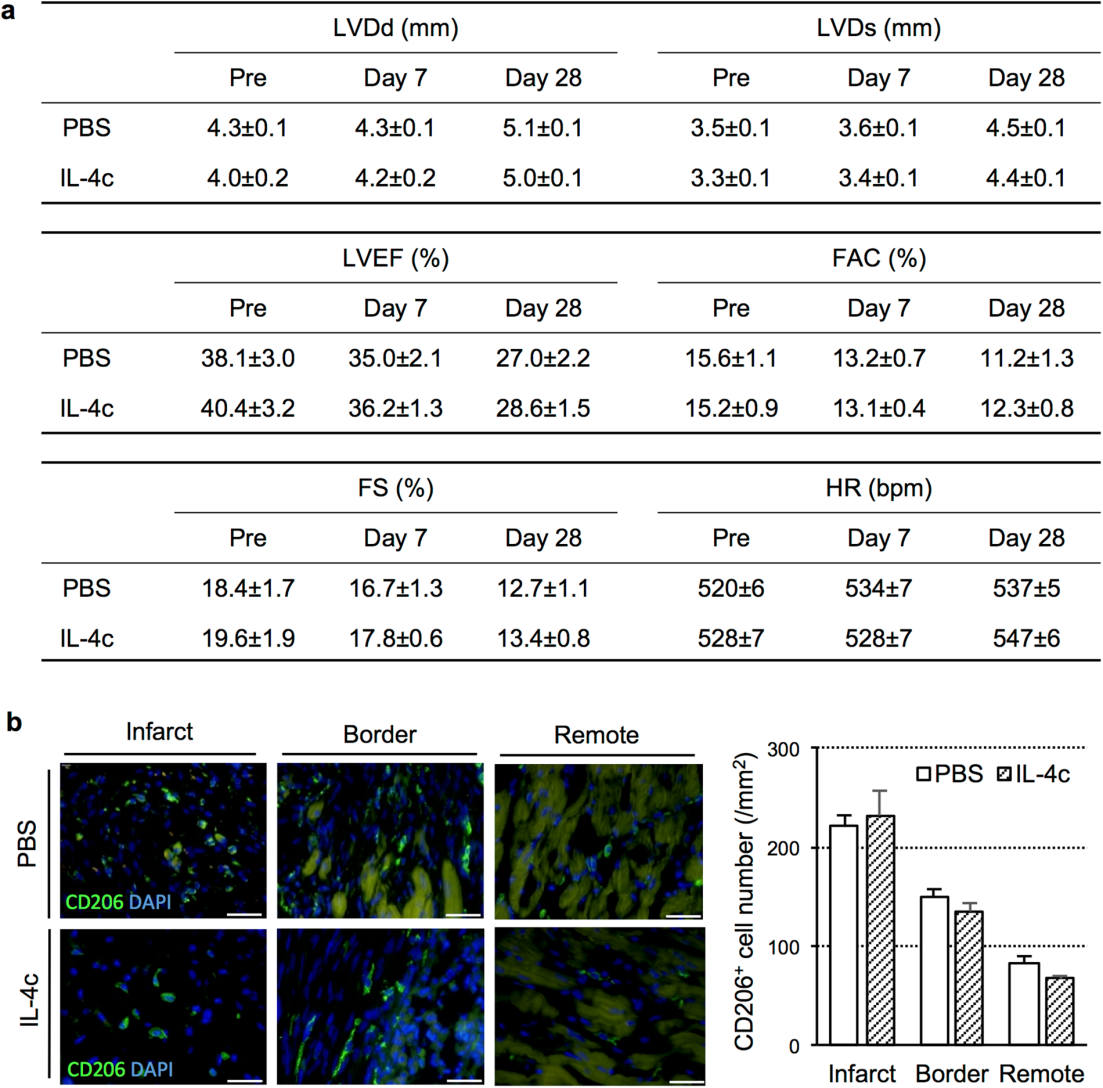
Ineffectiveness of IL-4c treatment for post-MI chronic heart failure. At 28 Days after coronary artery ligation, IL-4c (IL-4c group) or PBS (PBS group) was injected intraperitoneally to the mouse. (**a**) Changes in cardiac function and dimensions were assessed by echocardiography pre- and post-treatment (Day 7 and 28). *N* = 10 in each group. (**b**) The hearts at Day 7 after treatment were stained for CD206 with nuclear staining using DAPI. Scale bars, 100 μm. The numbers of CD206^+^ cells in each area of the MI heart were shown in the graph. *N* = 5 hearts in each group.

At a late phase post-MI (4 weeks after left coronary artery ligation) in mice, IL-4c or PBS (control) was injected. at Day 7 or 28 post-treatment, echocardiography revealed that cardiac function or structure in the IL-4c group was not improved compared to the PBS group (Fig. 8a). Pre-treatment echocardiography data were unchanged between the groups (Fig. 8a). Furthermore, the immunohistolabeling assessments demonstrated that the numbers of cardiac CD206^+^ cells in each infarct, border, and remote area at Day 7 post-treatment were unchanged between two groups (Fig. 8b). These data suggested that IL-4c administration was not able to increase M2-like macrophages in the chronically failing myocardium at a late phase post-MI and consequently failed to achieve therapeutic benefits to chronic ischemic heart failure.

## Discussion

This translational study demonstrated that injection of a long-acting form of IL-4 improved cardiac function and structure in a mouse model of the treatment of acute MI. The IL-4 administration after the onset of MI was able to increase CD206^+^F4/80^+^ M2-like macrophages, which highly expressed a range of anti-inflammatory and tissue repair-related genes, including *Il10, Il1rn, Hif1a, Vegfa, Igf1, Cxcl12, Spp1* and *Tgfb*, in the damaged myocardium. This resulted in the improved myocardial repair and attenuated adverse remodeling (*i.e*. local formation of denser and thicker connective tissues in the infarcted ventricular wall, increased microvascular formation in the viable myocardium and attenuated hypertrophy of surviving cardiomyocytes). Both systolic and diastolic ventricular function was improved, while cardiac dilatation was attenuated. These data suggest that IL-4 administration post-MI enabled to strengthen the intrinsic myocardial-repair system, in which cardiac M2-like macrophages play a role, highlighting a potential of IL-4 as a promising biological drug for the treatment of MI.

This article described that single injection of long-acting IL-4c achieved significant therapeutic benefits to acute MI. It is possible to develop a clinical-grade IL-4c (recombinant IL-4 mixed with stabilizing anti-IL-4 antibody); however, continuous or repeated infusion/injection of IL-4 protein only (without the antibody) may be able to reproduce the similar therapeutic effect with loser cost and less effort in the pre-clinical development, *i.e*. regulatory authorization. Controlled local delivery of IL-4, which will be obtained by using a biomaterial like hydrogels^28^, may be effective to achieve a continuous high dose of IL-4 in the heart. This would also reduce the necessary total dose of IL-4 compared to systemic injection, thereby decreasing the risk of its systemic side effects. Or, intracoronary injection at the time of percutaneous coronary intervention for acute MI may be another clinically applicable option, as this would offer a higher (and thereby longer-lasting) local concentration of IL-4 in the heart using the same dose of IL-4.

Several pieces of our results supported that myocardial repair induced by IL-4c treatment was achieved through enhancement of the cardiac M2 macrophage-mediated intrinsic myocardial-repair system, rather than through direct effects of IL-4 on other cardiac cells. Most importantly, in the *Trib1* ^*−/−*^ mice that showed a depleted ability to develop M2 macrophages, IL-4c treatment could not improve myocardial repair or cardiac function post-MI. In terms of the fibrotic tissue repair of the infarcted myocardium, co-culture with CD206^+^F4/80^+^ M2-like macrophages isolated from the infarcted heart markedly activated cardiac fibroblasts to form αSMA^+^ myofibroblasts, while an addition of up to 500 ng/ml IL-4 did not activate cardiac fibroblasts. In addition, expression of *Il4ra* in cardiac CD206^+^F4/80^+^ cells was 10^5^-fold greater than that of isolated primary cardiac fibroblasts. Also, it was confirmed that cardiac CD206^+^F4/80^+^ M2-like macrophages isolated from the post-MI heart expressed higher levels of major pro-fibrotic factors, *i.e*. TGFβ and Osteopoetin^2, 26^. These observations support that IL-4c-induced formation of more solid infarcts was achieved through cardiac M2-like macrophages rather than by direct effects of IL-4 on cardiac fibroblasts. Having said this, further focused investigations are required to elucidate molecular mechanism by which IL-4c treatment achieved cardiac repair post-MI.

There were reports suggesting possible adverse side effects of IL-4, including pathological fibrosis, atherosclerosis and exacerbation of allergic diseases like asthma^29–31^, raising concerns on its clinical application. However, previous results from the clinical trials of IL-4 injection to treat cancers^20–24^ ease such worries. These trials have reported that injection of a total dose of less than 200 μg/kg body weight IL-4 was well tolerated with no significant adverse effects. Furthermore, even though higher doses of IL-4 occasionally caused liver enzyme leakage, blood cell alterations, and edema, these were actually “non-critical”. In our study, 200 μg/kg IL-4 (5 μg IL-4 in a 25 g mouse) was sufficient to offer effective treatment for acute MI, and this dose could be reduced based on the result of a future dose-response study. Taken together, IL-4 treatment for acute MI is a repurposing application of a drug that has been proven clinically safe, ensuring the low-risk development of a highly valuable medicine.

To conclude, this study demonstrated that IL-4c administration at an acute phase of MI enhanced cardiac repair through enhancing the M2-like macrophage-involved self-repair mechanism. This represents preclinical evidence that IL-4 has great potential as a new (re-purposed) biological drug for the treatment of acute MI, encouraging the further development toward early clinical application.

## Methods

### Animals

All animal studies were performed with the approval of the ethics committee at the Queen Mary University of London and the UK Home Office (Project License; PPL70/7254). The investigation conforms to the Principles of Laboratory Animal Care formulated by the National Society for Medical Research and the Guide for the Care and Use of Laboratory Animals (US National Institutes of Health Publication, 1996), and all *in-vivo* procedures were carried out by UK Home Office Personal License holders. Male 10–12 weeks old C57BL/6 mice were purchased from Charles River Laboratories, UK. *Trib1* ^*−/−*^ mice were a gift from Professor Shizuo Akira (Osaka University, Japan) and were a hybrid of a C57BL/6 and SV129 mixed background^27^. Mice were maintained specific pathogen free in our animal facility on 12-hour light-dark cycle, with free access to food and water. Mice were randomly assigned to groups, and where possible, procedures and assessments were carried out in a blinded manner until statistical analysis.

### *In-vivo* procedures

MI was induced by ligation of the left coronary artery using an 8–0 polypropylene suture through left thoracotomy under 1.0% isoflurane anesthesia and mechanical ventilation^9, 32^. At 20 minutes or Day 28 after coronary artery ligation (a model for the treatment of acute MI or post-MI chronic heart failure, respectively), IL-4c (5 μg of recombinant mouse IL-4 [PeproTech, 214-14] mixed with 25 μg of a neutralizing monoclonal anti-IL-4 antibody [BD Biosciences, 554387] dissolved in 100 μl of phosphate-buffered saline [PBS]) was injected into the peritoneal cavity of the mice). IL-4c is known to have an expanded *in-vivo* half-life of IL-4 (over 24 hours *versus* 30 minutes for sole protein)^17, 25^. An equivalent volume of PBS was injected in a similar method as a control.

### Isolation of cardiac M2-like macrophages

Mouse cardiac M2-like macrophages were isolated by FACS as previously described^9^. Immediately after cervical dislocation, the aorta was clamped and cooled Hanks’ Balanced Salt solution (HBSS: Sigma-Aldrich) was injected into the LV cavity. The isolated hearts were cut into 1 mm^3^ pieces, digested by culturing with 0.05% collagenase II (Sigma-Aldrich) in HBSS at 37 °C for 15 minutes, and filtered using a 40-μm cell strainer (BD Falcon). The remnant heart tissues were again digested with a fresh digestion solution and filtered similarly; this cycle was repeated 5 times. The suspension obtained at each cycle was combined and subjected to erythrocyte depletion using Red Cell Lysis Buffer (BioLegend) according to the manufacturer’s protocol. The collected cells were re-suspended in FACS buffer (HBSS + 2 mM EDTA + 0.5% bovine serum albumin) and pre-incubated with an anti-mouse CD16/CD32 antibody (rat, 1:100 dilution, eBioscience, 14-0161) to block the Fc receptor. The dead cells and debris were excluded by forward scatter/side scatter and DAPI (1:1000 dilution, Sigma-Aldrich) staining. The cells were then labeled with PE-conjugated anti-F4/80 antibody (rat, 1:20 dilution, eBioscience, 12-4801) and AlexaFluor488-conjugated anti-CD206 antibody (rat, 1:50 dilution, BioLegend, 141709) and subjected to cell sorting using FACSAria II (BD Biosciences).

### Production and culture of heart slices

Viable heart slices were cut and cultured as described previously^33^. The mouse was euthanized and the hearts was immediately removed and placed into ice-cold Krebs solution (120 mM NaCl, 5 mM KCl, 1 mM CaCl_2_, 1.2 mM KH_2_PO_4_, 1 mM MgSO_4_, 25 mM NaHCO_3_, 11.5 mM glucose, pH 7.4). Slices (300 μm thick) were cut from these hearts using a vibratome (Energy Beam Sciences, Micro-Cut H1200) and quickly transferred to a 24 well plate to which just enough cold Krebs solution added to keep the slices wet. The plate was incubated at 37 °C for 20 minutes. Then, Krebs solution was removed and warm culture medium (DMEM with 10% Medium199, 10% horse serum, 5% FBS, 50 U/ml penicillin and 50 μg/ml streptomycin) with or without IL-4 (20 ng/ml) was added. The slices were cultured at 37 °C in a humid atmosphere with 5% CO_2_.

### Co-culture of cardiac fibroblasts and cardiac M2-like macrophages in a Boyden Chamber

Cardiac fibroblasts (2 × 10^4^) were plated on 0.1% gelatin-coated 6-well dish (Thermo Scientific) and cultured in RPMI1640 medium containing 10% FBS, 50 U/ml penicillin, and 50 μg/ml streptomycin. After 48 hours, 2 × 10^4^ of CD206^+^F4/80^+^ M2-like macrophages were seeded on polycarbonate membrane inserts (pore size 0.4 μm: Thermo Scientific), which were set into the well of the fibroblast culture^9^. At 48 hours of co-culture, fibroblasts were subjected to the analyses.

### Other standard procedures

Other widely-adopted research techniques used in this study (Echocardiography, Cardiac catheterization, Immunohistochemistry, Cell count using the histological samples, Picrosirius red staining, RNA extraction and real-time PCR, Isolation of cardiac fibroblasts, Immunocytochemistry) are described in the Supplementary Methods.

### Statistical analysis

All data are expressed as mean ± standard error of the mean (SEM), and statistical analysis was performed with Prism Software (version 6; GraphPad, CA, USA). For comparisons between multiple groups, one-way ANOVA (Figs 2a, 5g,h and 6b) or with one-way ANOVA with repeated measures (Fig. 8a) was performed followed by Bonferroni *post hoc* test. Comparisons between two groups were made by Student’s *t*-test. *P* < 0.05 was considered to be statistically significant.

## Electronic supplementary material

Supplementary Information
