# Supplementary material for: IL-4 as a Repurposed Biological Drug for Myocardial Infarction through Augmentation of Reparative Cardiac Macrophages: Proof-of-Concept Data in Mice
Source: Sci Rep. 2017 Jul 31;7:6877. doi: 10.1038/s41598-017-07328-z (PMC5537273; doi:10.1038/s41598-017-07328-z)
Supplement: Supplementary file 1 — Supplementary Information [file 41598_2017_7328_MOESM1_ESM.pdf]

SUPPLEMENTARY MATERIALS

IL-4 as a Repurposed Biological Drug for Myocardial Infarction through Augmentation of Reparative Cardiac Macrophages: Proof-of-Concept Data in Mice

Yusuke Shintani MD PhD, Tomoya Ito PhD, Laura Fields PhD, Manabu Shiraishi MD,

Yuki Ichihara MD PhD, Nobuhiko Sato MS, Mihai Podaru BS,

Satoshi Kainuma MD PhD, Hiroyuki Tanaka MD PhD, Ken Suzuki MD PhD

LIST OF SUPPLEMENTARY MATERIALS

- *Supplementary Methods*
- *Supplementary Fig. S1. Representative images of cardiac M2-like macrophages in the remote and border areas at Day 7 post-MI (Supplementary images to Fig. 1)*
- *Supplementary Fig. S2. Increased cardiac M2-like macrophages at Day 28 after IL-4 treatment*
- *Supplementary Fig. S3. Unchanged activation of fibroblasts in the remote and border areas after IL-4 treatment*
- *Supplementary Table S1. Echocardiography measurements at Day 28 in the acute MI model (Supplementary data to Fig. 4d)*
- *Supplementary Table S2. Cardiac catheterization data at Day 28 in the acute MI model (Supplementary data to Fig. 4e)*

SUPPLEMENTARY METHODS

Echocardiography. Transthoracic echocardiography was performed by using a Vevo-770 (VisualSonics/Fuji Film) with a 30 MHz high-frequency transducer under 1.0% isoflurane inhalation (9, 32). Left ventricular (LV) ejection fraction was calculated with the Simpson's method from the 2-dimensional tracing. LV end-diastolic and end-systolic dimensions were measured with the M-mode. LV end-diastolic and end-systolic areas were measured with the B-mode, from which fractional area change (FAC) was calculated. Data were collected from 4 different measurements per mouse in a blinded manner.

Cardiac catheterization. Hemodynamic parameters and cardiac function were also measured by using cardiac catheterization (9, 32). Briefly, under general anesthesia using isoflurane and mechanical ventilation, a catheter (SPR-839NR; Millar Instruments) was inserted into the LV cavity *via* the LV free wall through left thoracotomy. Intra-LV pressure signals were measured (MPVS-300; Millar Instruments) and digitally recorded with a data acquisition system (PowerLab 8/30; ADInstruments). The data were collected from at least 5 different measurements per mouse in a blinded manner.

Immunohistochemistry. Immediately after cervical dislocation, the aorta of the mouse was clamped under thoracotomy and ice-cold PBS with 16 mEq/l potassium was injected into the LV cavity using a 23G needle until the heart stopped beating. The heart was then perfused by injecting ice-cold 4% of paraformaldehyde in PBS into the LV cavity. Or, the viable heart slices were cultured with 4% of paraformaldehyde in PBS as described below. Then, the heart or heart slice was removed, cut, embedded in O.C.T. compound (VWR International) and frozen in isopentane chilled in liquid nitrogen. The frozen tissue sections (7 μm thick) were

cut from the heart or heart-slice samples and incubated in PBS containing 0.1% of Triton X (Sigma-Aldrich) for 5 minutes at the room temperature, and the non-specific antibody binding sites were pre-blocked with the blocking buffer (PBS plus 5% of goat serum). Samples were incubated with the following primary antibodies overnight at 4°C: AlexaFlour488-conjugated anti-CD206 (1:100 dilution BioLegend 141709), anti-Thy1 (1:100 dilution eBioscience 14-0901), anti-αSMA (1:100 dilution Abcam ab5694), anti-cleaved caspase-3 (1:200 dilution Cell Signaling 9661), anti-F4/80 (1:100 dilution Abcam ab6640), anti-wheat germ agglutinin (1:100 dilution ThermoFisher W11261), and anti-Ki67 (1:100 dilution eBioscience 14-5698). For isolectin B4 staining, biotinylated Griffonia simplicifolia lectin I-isolectin B4 (1:100 dilution Vector L-1104) was used. After rinsing in PBS 3 times for 5 minutes, the sections were next incubated with the following appropriate fluorophore-conjugated secondary antibodies were required: AlexaFluor 488-conjugated antibody (1:300 dilution Invitrogen A-11006) and AlexaFlour 594-conjugated antibody (1:300 dilution Invitrogen A-11007), together with 4',6-diamidino-2-phenylindole (DAPI) for nuclear counterstaining, in the blocking buffer for 1 hour at the room temperature. Stained sections were mounted with DAKO Fluorescence Mounting Medium and the digital images were acquired with an All-in-One microscope (Keyence, UK).

Cell count using the histological samples. To assess the number of positively stained cells, only cells that were clearly stained and have nuclei (DAPI stained) were counted in each area. The infarct area was defined as the LV free wall myocardium in which > 90% of cardiomyocytes were lost, and the border area was defined as an adjacent area to the infarct area with approximately 50% of cardiomyocytes being alive. Remote area was non-ischemic myocardium in the ventricular septum. More than 6 fields for each area were assessed per heart.

Picrosirius red staining. Seven-μm frozen sections were incubated in 1.5% of phosphomolybdic acid for 60 minutes, next in 0.1% of Picrosirius red for 15 minutes, and then in 0.5% of acetic acid solution for 3 minutes. After dehydration through increasing concentrations of ethanol to xylene, the sections were mounted using the DPX mounting medium (VWR International). The infarct size (the ratio of scar length to total left ventricular circumference) and the wall thickness were measured at five independent regions of the infarct area as previously described (9, 32). The quantity of the collagen fraction was calculated from 6 fields of each area per mouse by using a National Institute of Health image-analysis software (ImageJ).

RNA extraction and real-time PCR. Total RNA was extracted from cells or LV free walls (including the infarct and border areas) using the Gene Jet PCR purification Kit (Thermo Scientific) and quantified with a Nano-Drop 8000 spectrophotometer (Thermo Scientific). cDNA was synthesized from 25 ng and 150 ng of total RNAs from M2 macrophages and the heart tissues, respectively, by using the high-capacity cDNA Reverse Transcription Kit (Applied Biosystems). Real-time PCR was performed by a 7900HT (Applied Biosystems) with a SYBR Green I master mix (Roche) in following conditions: 95°C for 10 minutes followed by 50 cycles at 95°C for 15 seconds, 64°C for 30 seconds and 72°C for 30 seconds. Gene expression levels were normalized by *Gapdh*. The primers used are:

Il10	forward	5’- CGCTGTCATCGATTTCTCC -3’
	reverse	5’- ACACCTTGGTCTTGGAGCTT -3’
Vegfa	forward	5’- GTACCTCCACCATGCCAAGT -3’
	reverse	5’- GCATTCACATCTGCTGTGCT -3’
Spp1	forward	5’- GATAGCTTGGCTTATGGACTGAGGT -3’
	reverse	5’- GACTCCTTAGACTCACCGCTCTT -3’
Il1rn	forward	5’- GTGCCTATTGACCTTCATAGTGTGTTC -3’
	reverse	5’- GCGCTTGTCTTCTTCTTTGTTCT -3’
Col1a1	forward	5’- TGAGCCAGCAGATTGAGAAC -3’

	reverse	5’- CCAGTACTCTCCGCTCTTCC -3’
Col3a1	forward	5’- AGTCTGGAGTCGGAGGAATG -3’
	reverse	5’- AGGATGTCCAGAGGAACCAG -3
Mrc1 (CD206)	forward	5’- ACTACACACTCATCCATTACAACCAA -3’
	reverse	5’- GGCACCTATCACAATCAGGAGGA -3’
Retnla	forward	5’- AGGAACTTCTTGCCAATCCA -3’
	reverse	5’- ACAAGCACACCCAGTAGCAG -3’
Chil3	forward	5’- TGGTGAAGGAAATGCGTAAA -3’
	reverse	5’- GTCAATGATTCCTGCTCCTG -3’
Hprt	forward	5’- AGCGATGATGAACCAGGTTA -3’
	reverse	5’- GTTGAGAGATCATCTCCACC -3’
Gapdh	forward	5'- TAGACAAAATGGTGAAGGTCGGTGT -3'
	reverse	5'- AATGAAGGGGTCGTTGATGG -3'
Hif1a	forward	5'- TCAGCATACAGTGGCACTCA -3'
	reverse	5'- GGTTAAGGCTCCTTGGATGA -3'
Il1b	forward	5’- TCTATACCTGTCCTGTGTAATGAAAGAC -3’
	reverse	5’- CACTTTGCTCTTGACTTCTATCTTGTTG -3’
Tnfa	forward	5’- AGCCTCTTCTCATTCCTGCTTGT -3’
	reverse	5’- GTTTGTGAGTGTGAGGGTCTGG -3’
Tgfb	forward	5'- CAACTTCTGTCTGGGACCCT -3'
	reverse	5'- CGGGTTGTGTTGGTTGTAGA -3'
Igf1	forward	5’- GGACCGAGGGGCTTTTACTTC -3’
	reverse	5’- GGCACAGTACATCTCCAGTCTCCTC -3’
Cxcl12	forward	5’- ATCTGAAAATCCTCAACACTCCAAAC -3’
	reverse	5’- GATCCACTTTAATTTCGGGTCAA -3’

Isolation of cardiac fibroblasts. Cardiac fibroblasts were isolated from 200-250 g male Sprague-Dawley rats (Charles River Laboratories) as described previously (9). Isolated rat heart was cut into 1 mm^3 pieces, and the fragments were plated evenly in 0.1% gelatin-coated 10-cm dishes not to contact each other. Each fragment was covered by a droplet of RPMI1640 medium containing 10% FBS, 50 U/ml penicillin and 50 μg/ml streptomycin, and incubated for 24 hours in a CO_2 incubator. Then, the minimum volume of medium was added to cover the whole bottom surface of culture dish, and the culture was continued for an additional 24 hours. Sufficient medium was then added, which was cultured for additional 5

days. When the outgrowth of fibroblast was observed, cells were collected by trypsinization. Collected fibroblasts were plated in 0.1% gelatin-coated culture flask and maintained with RPMI1640 containing 10% FBS, 50 U/ml penicillin, and 50 μg/ml streptomycin with or without ranging (0-500 ng/ml) IL-4 (PeproTech 214-14). Cells were used at passage 3 or 4.

Immunocytochemistry. The cultured cells were fixed by 4% of paraformaldehyde in PBS for 5 minutes at room temperature. The cells were incubated in PBS containing 0.1% of Triton x100 for 5 minutes at the room temperature. Non-specific antibody binding sites were pre-blocked with PBS containing 5% of goat serum for 30 minutes at the room temperature. Then primary antibodies, anti-vimentin (1:100, Abcam, ab24525) and anti-αSMA (1:100, Abcam, ab5694), were applied onto the cells for 1 hour at room temperature. After rinsing, cells were incubated with the fluorophore-conjugated secondary antibodies (1:300, AlexaFluor 488-conjugated and AlexaFluor 594-conjugated polyclonal, Invitrogen A11039, A-21208, and A-11037) and DAPI in blocking buffer for 1 hour at room temperature. Stained cells were mounted with DAKO Fluorescence Mounting Medium and the digital images were acquired with an All-in-One microscope (Keyence, UK).

SUPPLEMENTARY FIGURE S1

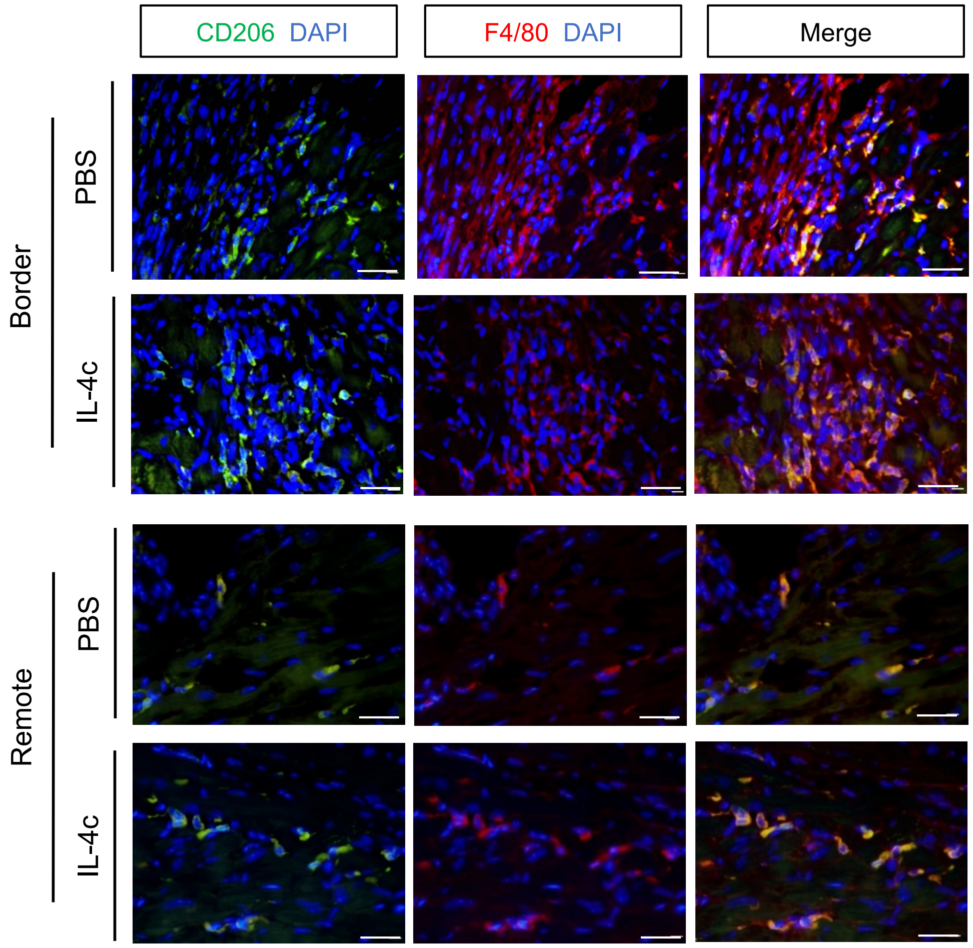

Supplementary Fig. S1. Representative images of cardiac M2-like macrophages in the remote and border areas at Day 7 post-MI (Supplementary images to Fig. 1)

Following coronary artery ligation, IL-4c (IL-4 group) or control PBS (PBS group) was injected intraperitoneally to the mouse. The hearts were stained for CD206 and F4/80 with nuclear staining using DAPI. Representative pictures from the border and remote areas in each group were presented. The numbers of positive cells in each area were counted and shown in Fig. 1B-D. Scale bars, 100 μm.

SUPPLEMENTARY FIGURE S2

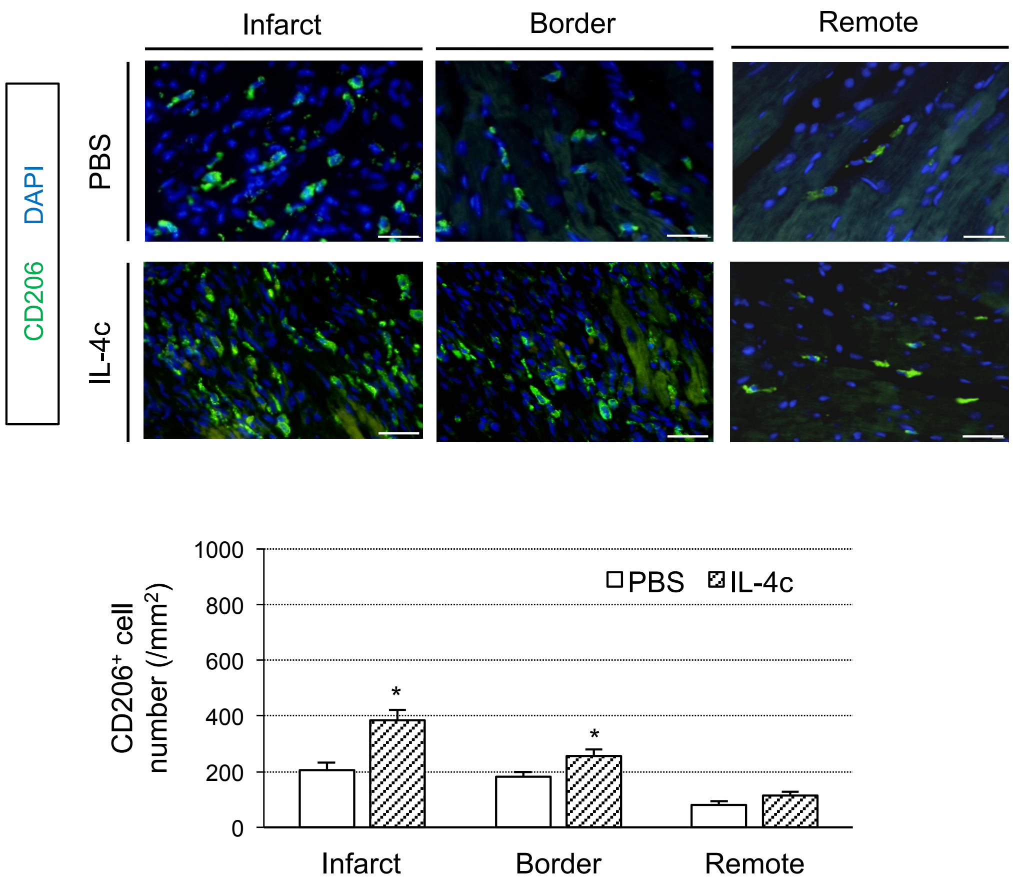

Supplementary Fig. S2. Increased cardiac M2-like macrophages at Day 28 after IL-4 treatment

Following coronary artery ligation, IL-4c (IL-4 group) or control PBS (PBS group) was injected intraperitoneally to the mouse. At Day 28 after treatment (IL-4c or PBS injection), the hearts were collected and stained for CD206 with DAPI nuclear staining. Scale bars, 100 μm. The numbers of $CD206^{+}$ cells in each area of the MI heart were shown in the graph. N=5 in each group; *P<0.05 versus the PBS group.

SUPPLEMENTARY FIGURE S3

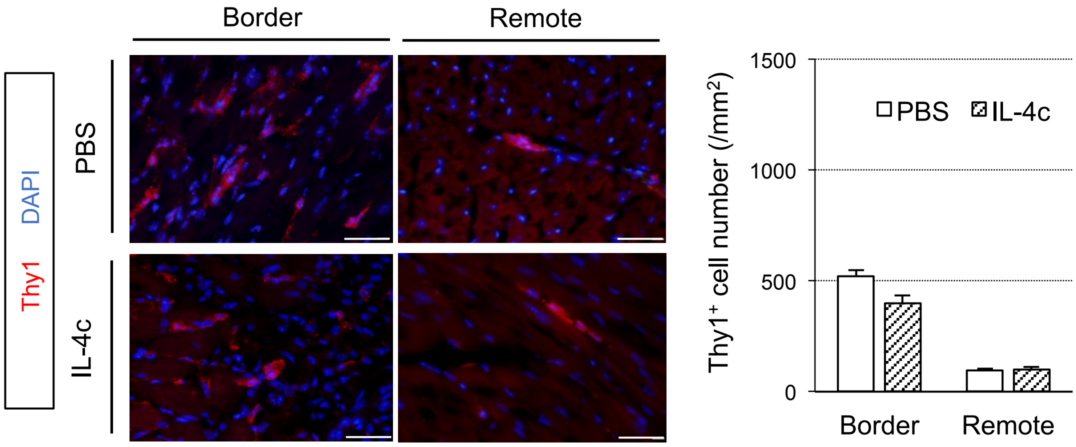

Supplementary Fig. S3. Unchanged cardiac fibroblasts in the remote and border areas after IL-4 treatment

The hearts at Day 7 after treatment (IL-4c or PBS injection) in each group were stained for Thy1 with DAPI nuclear staining. Scale bars, 50 μm. The number of $Thy1^+$ cells in the border and remote areas were counted and shown in the graph. For the data in the infarct area, please see Fig. 5 A-D. *N*=5 different hearts in each group.

SUPPLEMENTARY TABLE S1

	LVDd (mm)	LVDs (mm)	LVFS (%)
PBS	4.7 ± 0.2	3.9 ± 0.2	18.3 ± 0.4
IL-4c	4.1 ± 0.1*	3.3 ± 0.2*	23.1 ± 0.5*

	LVEAD (mm^2)	LVEAS (mm^2)	LVFAC (%)
PBS	24.5 ± 1.7	22.8 ± 0.6	12.2 ± 0.8
IL-4c	15.7 ± 0.4*	10.9 ± 0.1*	17.2 ± 0.6*

	LVEF (%)	HR (bpm)	*n*
PBS	38.0 ± 1.0	514.2 ± 3.2	11
IL-4c	46.6 ± 0.8*	519.1 ± 4.0*	11

Supplementary Table S1. Echocardiography measurements at Day 28 in the acute MI model (Supplementary data to Fig. 4d)

PBS group; PBS injection group, IL-4c group; IL-4c treatment group, LVDd; left ventricular diastolic dimension, LVDs; left ventricular systolic dimension, LVEAD; left ventricular diastolic endocardial area, LVEAS; left ventricular systolic endocardial area, LVEF; left ventricular ejection fraction, LVFAC; left ventricular fractional area change, LVFS; left ventricular fractional shortening, HR; heart rate. *P<0.05 versus the PBS group.

SUPPLEMENTARY TABLE S2

	Max Pressure (mmHg)	Min Pressure (mmHg)	Mean Pressure (mmHg)
PBS	104.6 ± 8.0	6.1 ± 0.6	55.8 ± 4.9
IL-4c	117.7 ± 1.7	1.7 ± 0.3*	50.6 ± 1.5

	Dev. Pressure (mmHg)	LVEDP (mmHg)	Systolic duration (ms)
PBS	98.6 ± 7.6	7.4 ± 0.7	83.2 ± 5.7
IL-4c	116.1 ± 1.9*	3.6 ± 0.4*	65.9 ± 0.7*

	Diastolic duration (ms)	Max dP/dt (mmHg/s)	Min dP/dt (mmHg/s)
PBS	54.2 ± 5.9	4356 ± 388	-2462 ± 130
IL-4c	70.8 ± 1.8*	6558 ± 411*	-6429 ± 723*

	IRP Average dP/dt (mmHg/s)	Tau (s)	Pressure Time Index (mmHg·s)
PBS	-1648 ± 180	0.0302 ± 0.0036	6.4 ± 0.7
IL-4c	-3443 ± 495*	0.0202 ± 0.0042	5.6 ± 0.3

	Heart Rate (bpm)	*n*
PBS	430.8 ± 6.3	6
IL-4c	440.1 ± 7.2	6

Supplementary Table S2. Cardiac catheterization data at Day 28 in the acute MI model (Supplementary data to Fig. 4e)

PBS group; PBS injection group, IL-4c group; IL-4c treatment group, LVEDP; left ventricular end-diastolic pressure, Dev. Pressure; developed left ventricular pressure, IRP; isovolumic relaxation period, Tau; left ventricular diastolic time constant. *P<0.05 versus the PBS group.
